# Electron-Beam Radiation Crosslinking as a Route for Upgrading Recycled Polyethylene for Circular Economy Applications

**DOI:** 10.3390/polym18141719

**Published:** 2026-07-13

**Authors:** Lyazat Tolymbekova, Gaini Seitenova, Aiymzhan Kazbekova, Aisha Baktybek, Murat Kassymzhanov, Eldar Kopishev, Zarina Yelemessova

**Affiliations:** 1 Department of Chemistry, Institute of Natural Sciences, L.N. Gumilyov Eurasian National University, Astana 010000, Kazakhstan; tolymbekova_lb@enu.kz (L.T.); seitenova_gzh@enu.kz (G.S.); aiymzhanr2502@gmail.com (A.K.); aishabaktybek1@gmail.com (A.B.); 2 Department of Industrial Production, JSC “Park of Nuclear Technology”, Kurchatov 071100, Kazakhstan; kasymzhanov@pnt.kz

**Keywords:** recycled polyethylene, electron-beam irradiation, polymer upgrading, radiation crosslinking, circular economy, gel fraction, thermal behavior, mechanical properties, structure–property relationship

## Abstract

The growing demand for polymer recycling requires effective approaches to improve the performance of recycled materials and expand their practical applications. In this study, electron-beam irradiation was investigated as a method for modifying recycled polyethylene obtained from façade-fastening elements. Virgin PE-80 polyethylene was used as a reference material for comparison. Irradiation was carried out using an ILU-10 electron accelerator (5 MeV) at doses of 95–125 kGy. Structural, morphological, elemental, thermal, crosslinking, and mechanical characteristics were evaluated using FTIR spectroscopy, SEM/EDS analysis, differential scanning calorimetry (DSC), gel fraction determination, and tensile testing according to ISO 527. The results showed that irradiation promotes the formation of a crosslinked network structure in both materials, as confirmed by the increase in gel fraction with increasing dose. For recycled polyethylene, gel fraction values increased from 46.7 to 56.2%, indicating effective radiation-induced crosslinking despite the structural heterogeneity of the material. FTIR analysis revealed the formation of oxygen-containing functional groups associated with radiation-induced oxidation, which was more pronounced in recycled polyethylene due to the presence of pre-existing defects and degradation products. SEM observations revealed increased surface roughness and localized fibrillar features after irradiation, while DSC analysis indicated a decrease in the crystallinity of recycled polyethylene associated with radiation-induced crosslinking and restricted molecular chain rearrangement. Mechanical testing showed an increase in tensile strength and elastic modulus accompanied by a reduction in elongation at break. Among the investigated irradiation doses, 110 kGy provided the most favorable balance between crosslinking efficiency and preservation of structural integrity. These findings demonstrate that electron-beam irradiation is an effective strategy for upgrading recycled polyethylene by improving its mechanical performance while maintaining structural integrity, thereby expanding its potential for reuse in circular economy applications.

## 1. Introduction

The development of the polymer industry is accompanied by a steady increase in the production of polymeric materials, which currently exceeds 400 million tons per year, while the share of recycling remains less than 20% [1,2,3]. This leads to the accumulation of significant volumes of polymer waste and the formation of serious environmental problems, necessitating the development of rational technologies for their processing and reuse [4,5].

Recycled polymeric materials are considered an important element of the circular economy concept; however, their practical application is limited by the deterioration of their performance properties compared to primary polymers [3,6,7,8]. During polymer processing, thermo-oxidative degradation, shortening of macromolecular chains, changes in molecular weight distribution, and the accumulation of structural defects occur, leading to a decrease in the mechanical strength, thermal stability, and durability of materials [5,6,9].

One promising method for modifying polymeric materials is radiation treatment, which allows for targeted changes in their structure without the introduction of chemical reagents [10,11,12]. Under the influence of ionizing radiation (γ-quanta or accelerated electrons), macroradicals are formed in the polymer matrix, which can participate in both interchain crosslinking reactions and chain scission reactions [10,13].

Radiation-induced crosslinking leads to the formation of a three-dimensional spatial network, which is accompanied by increased thermal stability, increased strength and elastic modulus, and decreased material solubility [14,15,16,17]. However, at high irradiation doses or in the presence of structural defects and impurities, radiation-induced degradation processes may intensify, leading to a deterioration in properties [5,18].

Among recycled polymers, polyethylene represents one of the largest waste streams due to its extensive use in packaging, construction, and engineering applications [1]. However, repeated processing and service exposure lead to thermo-oxidative degradation, structural defects, and deterioration of mechanical performance, limiting the reuse of recycled polyethylene in demanding applications [19]. Therefore, the development of effective post-processing technologies capable of restoring or improving the properties of recycled polyethylene is of considerable practical and environmental importance [20].

The use of electron-beam irradiation is a technologically feasible method for crosslinking polyethylene, offering several advantages, including high processing speed, the ability to precisely control the dose, and the absence of the need for initiators [11,21]. It has been shown that in the dose range of 50–150 kGy, a significant increase in the strength and thermal properties of polyethylene occurs due to an increase in cross-linking density [22,23].

At the same time, the behavior of secondary polymers under radiation exposure has been insufficiently studied. The presence of oxidation products, residual additives, impurities, and structural defects has a pronounced effect on the kinetics of radiation-induced processes and can lead to increased competition between cross-linking and degradation [4,5,18].

Modern methods of physicochemical analysis, such as infrared spectroscopy (FTIR), scanning electron microscopy (SEM), differential scanning calorimetry (DSC), and gel fraction determination [16,24,25,26], are widely used to study structural transformations in polymers. The combined application of these techniques enables correlations between molecular structure, morphology, and material properties to be established.

In a broader context, the formation of polymer networks and intermolecularly organized structures is an important factor controlling the functional behavior of polymeric materials. Previous studies on the complexation of interpolymers between cellulose ethers, poloxamers, and polyacrylic acid have shown that surface-dependent interactions strongly influence the organization and properties of polymer systems [27]. Network formation principles have also been explored in polyelectrolyte hydrogel-based materials and polymer systems intended for optical sensor applications, where the structural organization of polymer chains determines the functional response of the material [28,29]. Furthermore, studies of phase transitions in solutions of thermoresponsive polymers and hydrophilic polymer systems show that phase behavior, supramolecular organization, and intermolecular interactions play important roles in controlling polymer properties [30,31]. These results confirm the general importance of structure–property relationships in polymer systems and provide a broader conceptual framework for interpreting radiation-induced crosslinking and structural transformations in polyethylene materials.

Despite extensive research on radiation crosslinking of virgin polyethylene, the response of recycled polyethylene to electron-beam irradiation remains insufficiently understood. The heterogeneous composition of recycled polyethylene, together with the presence of residual degradation products and impurities, may significantly influence the balance between crosslinking and radiation-induced degradation. As a result, the mechanisms governing structure–property relationships in irradiated recycled polyethylene remain insufficiently clarified, limiting its broader application in high-value polymer products and circular economy technologies.

Therefore, the aim of this study was to evaluate the effectiveness of electron-beam radiation crosslinking as a method for upgrading recycled polyethylene obtained from façade-fastening elements. Virgin PE-80 polyethylene was used as a reference material to compare structural, thermal, morphological, and mechanical changes induced by irradiation. The novelty of this work lies in the comprehensive comparative evaluation of virgin and recycled polyethylene subjected to electron-beam irradiation, providing new insights into the influence of structural heterogeneity on crosslinking, oxidation, and property development.

## 2. Materials and Methods

### 2.1. Materials

FIXPLAST façade plate dowels supplied by STONEX (Astana, Kazakhstan) were used as the source of the recycled polymer material. The investigated material originated from post-industrial façade-fastening elements and was supplied as a commercial recycled product. Therefore, its exact quantitative polymer composition was not available.

FTIR analysis confirmed that polyethylene constitutes the dominant polymer matrix of the recycled material. The broader melting peak observed by DSC and the elemental composition revealed by EDS indicate the presence of minor additives, fillers, and residual components typical of recycled industrial plastics. These features result in greater structural and compositional heterogeneity compared with virgin polyethylene. Therefore, the investigated material was considered a polyethylene-based recycled thermoplastic throughout this work.

The use of the selected recycled polymer material made it possible to evaluate the effect of electron-beam radiation crosslinking under conditions representative of industrial applications and to assess the influence of structural heterogeneity on the relationship between polymer structure, radiation-induced crosslinking, and material properties.

The primary polymer material used in the work was high-density polyethylene (HDPE) grade PE-80, widely used to produce pressure pipes for water supply systems. The material was obtained from industrial-grade polyethylene pipes provided by LLP Politech (Astana, Kazakhstan). According to ISO 12162 [32], this material belongs to the PE-80 class with a minimum long-term strength (MRS) of 8.0 MPa [32]. Performance requirements for polyethylene-based pipes comply with ISO 4427 [33].

The material’s key physicochemical properties were determined according to international standards: density according to ISO 1183-1 [34], melt flow index according to ISO 1133-1 [35], and mechanical properties according to ISO 527-1 [36].

### 2.2. Sample Preparation

Before radiation treatment and mechanical testing, the source materials were subjected to pre-treatment. Polyethylene pipes and façade dowels were crushed using a laboratory-scale XHS-39E-C polymer crusher (Jinan Xinghua Instruments Co., Ltd., Jinan, China), which mechanically crushes the material into granules to produce a homogeneous raw material and improve the reproducibility of subsequent molding.

The obtained granulated material was hot-pressed using a laboratory-scale XHS-02QH hydraulic heat press (Jinan Xinghua Instruments Co., Ltd., Jinan, China). Compression molding was carried out at 185 °C, 8 MPa, and 7 min. After molding, the samples were cooled under pressure to room temperature to minimize internal stresses and prevent deformation. The resulting sheets had dimensions of 150 × 150 mm and a thickness of approximately 2 mm. Type 1A tensile specimens were then punched from the molded sheets in accordance with ISO 527-2:2012 [37]. The prepared specimens were used for subsequent radiation treatment, thermal analysis, and mechanical testing.

**Figure 1:**
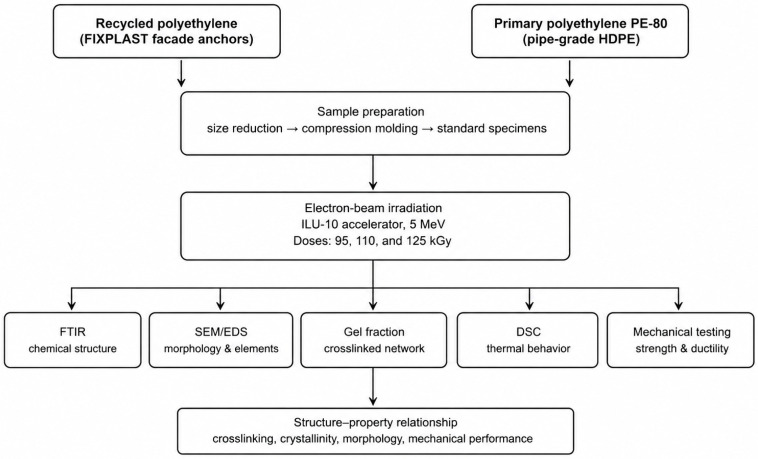
Schematic representation of the experimental workflow, including sample preparation, electron-beam irradiation using an ILU-10 (Institute of Nuclear Physics, Novosibirsk, Russia), and subsequent characterization of recycled polyethylene and reference PE-80 polyethylene by FTIR, SEM/EDS, gel fraction, DSC, and mechanical testing.

Figure 1 shows the general experimental process.

### 2.3. Radiation Crosslinking

Radiation crosslinking of recycled polyethylene and reference PE-80 polyethylene samples was performed at the Nuclear Technology Park (Kurchatov, Kazakhstan) using an ILU-10 pulsed linear electron accelerator with a maximum energy of 5 MeV.

Irradiation was performed at absorbed doses of 95, 110, and 125 kGy at room temperature in air. Samples were transported through the irradiation zone using a conveyor system, ensuring uniform dose distribution across the surface.

Beam parameters included an average power of up to 50 kW and a current of approximately 10 mA. Pulse duration was 0.4–0.5 ms. The selected electron energy ensured sufficient radiation penetration depth for uniform treatment of the samples across their entire surface.

After irradiation, the samples were kept at room temperature before further testing.

### 2.4. FTIR Analysis

Infrared spectra of the samples were recorded using an IRTracer-100 spectrometer (Shimadzu, Kyoto, Japan) in the wavenumber range of 4000–400 cm^−1^ at room temperature. Measurements were performed in ATR mode with a resolution of 4 cm^−1^ and accumulation of 32 scans for each sample. Spectral acquisition and processing were performed using LabSolutions IR software (version 2.21, Shimadzu, Kyoto, Japan).

The method was used to analyze changes in the chemical structure of recycled polyethylene and reference PE-80 polyethylene before and after radiation exposure.

Particular attention was paid to identifying characteristic absorption bands corresponding to the stretching and deformation vibrations of methylene groups (–CH_2_–), as well as the appearance of bands associated with oxygen-containing functional groups, including carbonyl (C=O) and hydroxyl (–OH) groups, the formation of which is due to radiation-induced oxidation processes [5,8].

A comparative analysis of the spectra of the original and irradiated samples was carried out to identify dose-dependent changes associated with the processes of interchain crosslinking and partial destruction of the polymer matrix.

### 2.5. SEM Analysis

The surface morphology and fracture structure of polymer samples before and after radiation crosslinking were examined using scanning electron microscopy (SEM) using a TM4000 microscope (Hitachi High-Tech, Tokyo, Japan).

Prior to analysis, the samples were fractured to obtain a representative fracture surface. Cryogenic fracture in liquid nitrogen was used to preserve the internal structure of the material and minimize plastic deformation. Due to the low vacuum mode of the TM4000 microscope, additional conductive coating was not required.

The studies were conducted at an accelerating voltage of 10–15 kV. Micrographs were obtained at a magnification of ×1000, chosen as optimal for analyzing surface morphology, structural heterogeneity, and the presence of defects.

The main focus of the analysis was on identifying structural differences between primary and secondary polymers, including the degree of matrix homogeneity, the presence of inclusions and defects, phase heterogeneity, and changes in destruction caused by radiation crosslinking [11,15].

### 2.6. Gel Fraction Determination

The degree of radiation crosslinking of recycled polyethylene and reference PE-80 polyethylene samples was assessed based on the gel fraction content by extraction of the soluble portion of the polymer in toluene using a Soxhlet apparatus, in accordance with methods widely used for the analysis of crosslinked polyolefins [38].

This method is based on the removal of soluble components, including non-crosslinked macromolecules and low-molecular-weight products of radiation degradation, followed by determination of the proportion of insoluble residue corresponding to the spatially crosslinked polymer network.

Samples weighing ~0.3–0.5 g were placed in cellulose extraction thimbles and subjected to continuous extraction in boiling toluene in a Soxhlet apparatus for 24 h at a temperature of ~110 °C. Toluene was used as a solvent, as it readily dissolves non-crosslinked polyethylene fractions without disrupting the crosslinked network structure.

After extraction, the samples were removed, dried in a drying oven at 70 °C for 12 h to constant weight, and cooled in a desiccator to room temperature. Weighing was performed on an analytical balance with an accuracy of ±0.001 g.

The gel fraction content (G, %) was calculated using the formula:

G (%) = (m_2_/m_1_) × 100

where

*G*—is the gel fraction content (%);
*m*_1_—is the initial dry mass of the sample before extraction (g);
*m*_2_—is the dry mass of the insoluble residue after Soxhlet extraction and drying (g).

Each measurement was performed in triplicate (*n* = 3). The results were expressed as mean values ± standard deviation (SD).

Gel fraction determination was used to quantify the degree of spatial network structure formation and analyze the effect of radiation dose on interchain cross-linking processes in the studied materials.

### 2.7. DSC Analysis

The thermal properties of recycled polyethylene and reference PE-80 polyethylene samples before and after radiation treatment were studied using differential scanning calorimetry (DSC) on a TA Instruments Q2000 calorimeter (TA Instruments, New Castle, DE, USA) in accordance with international standards ISO 11357-1 [39] and ISO 11357-3 [40]. Thermal data acquisition and analysis were performed using TRIOS software (version 5.2, TA Instruments, New Castle, DE, USA).

Samples weighing 6–8 mg were placed in sealed aluminum crucibles and analyzed under a nitrogen atmosphere (flow rate ~50 mL/min) to prevent oxidation during the experiment.

Measurements were conducted over a temperature range of 30 to 200 °C at a heating rate of 10 °C/min. To eliminate the thermal history of the material, a two-cycle mode was used: an initial heating, subsequent cooling to 30 °C, and a second heating. Thermal characteristics were analyzed using the second heating data.

Melting points (Tm) and enthalpies of melting (ΔHm) were determined from the endothermic peaks of the DSC curves. The degree of crystallinity (Xc, %) was calculated using the formula:

(1) $$X_{c}=\frac{\Delta H_{m}}{\Delta H_{m}^{0}}\times 100$$

where

$\Delta H_{m}$—the experimentally determined enthalpy of melting, J/g;
$\Delta H_{m}^{0}=293$ J/g—the enthalpy of melting of fully crystalline polyethylene [41].

The obtained data were used to analyze the influence of radiation crosslinking on the degree of crystallinity and thermal behavior of the studied polymeric materials.

Due to experimental limitations, DSC analysis was performed for the initial samples and for samples irradiated at 110 kGy, which was selected as a representative dose corresponding to the region of intensive radiation-induced structural transformations, as confirmed by gel fraction and mechanical property changes.

### 2.8. Mechanical Testing

The mechanical properties of recycled polyethylene and reference PE-80 polyethylene samples before and after radiation treatment were determined using the uniaxial tensile test method in accordance with ISO 527-2:2012 [37].

The tests were conducted on an XH Instruments Testing System universal tensile testing machine (Jinan Xinghua Instruments Co., Ltd., Jinan, China), an electronic-mechanical testing system equipped with a load cell and a computerized data recording system.

The samples were shaped as Type 1A blades in accordance with ISO 527-2 [37]. The gauge length was 50 mm, and the thickness of the samples was approximately 2 mm.

The tests were conducted at room temperature (23 ± 2 °C) with a crosshead speed of 50 mm/min, which corresponds to standard polyethylene testing conditions.

Five specimens were tested for each material condition (initial and irradiated at different doses). During the tensile tests, tensile strength, elongation at break, and elastic modulus were determined.

The experimental results are presented as mean values ± standard deviation (SD) calculated from five independent measurements. Statistical processing of the experimental data was performed using Microsoft Excel 2021 (Microsoft Corporation, Redmond, WA, USA).

Mechanical testing was used to evaluate the effect of radiation crosslinking on the deformation behavior of the materials and to establish the relationship between structural changes and the performance characteristics of the polymers.

## 3. Results

### 3.1. FTIR Results

**Figure 2:**
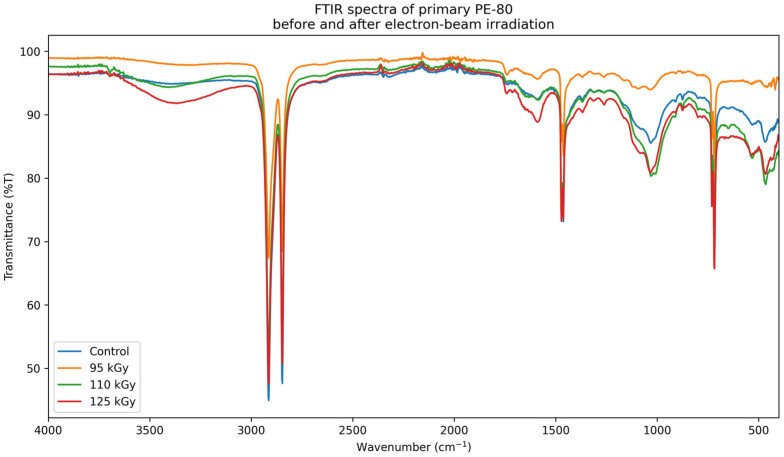
FTIR spectra of virgin PE-80 before irradiation and after electron-beam irradiation at absorbed doses of 95, 110, and 125 kGy.

**Figure 3:**
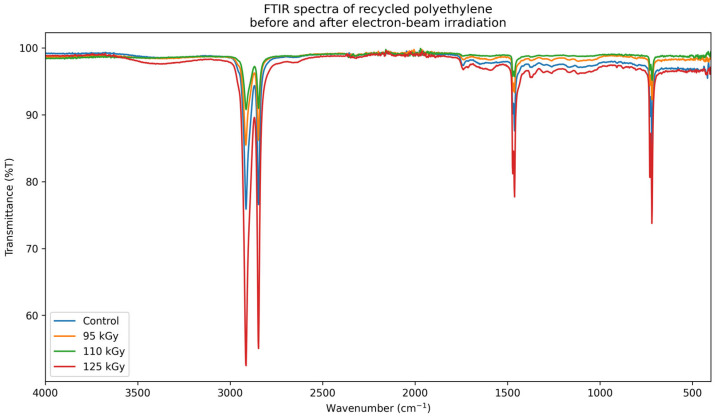
FTIR spectra of recycled polyethylene before irradiation and after electron-beam irradiation at absorbed doses of 95, 110, and 125 kGy.

Figure 2 and Figure 3 show the FTIR spectra of PE-80 polyethylene and recycled polyethylene before and after electron-beam irradiation at doses of 95, 110, and 125 kGy. PE-80 was used as a comparison material with a more regular structure, which makes it possible to distinguish the spectral features associated with the heterogeneous nature of the recycled polymer. The spectra are presented in wavenumber–transmittance (%T) coordinates and were used to evaluate dose-dependent changes in the chemical structure of both materials.

The spectrum of the PE-80 control sample shows characteristic absorption bands of polyethylene: intense bands in the 2915–2920 and 2848–2850 cm^−1^ regions, corresponding to the asymmetric and symmetric stretching vibrations of the –CH_2_– methylene groups; a band at approximately 1470 cm^−1^, associated with the deformation vibrations of the –CH_2_– groups; and peaks in the 720–730 cm^−1^ region, caused by the rocking vibrations of the long methylene sequences of the polyethylene chain. The presence of these bands confirms the preservation of the basic hydrocarbon structure of the polyethylene matrix.

After electron-beam irradiation, the main polyethylene bands are retained in all samples, indicating that the PE-80 macromolecular chain is not completely destroyed. At the same time, changes in the intensity of individual bands are observed, particularly in the 1700–1740 cm^−1^ and 3200–3500 cm^−1^ regions, which may be related to the formation of oxygen-containing functional groups associated with radiation-induced oxidation.

For primary PE-80, changes in the region of oxygen-containing groups are moderate compared to recycled polyethylene. This can be explained by the higher structural homogeneity of the virgin material and the lower content of initial defects, thermal-oxidative degradation products, and foreign impurities. Increasing the irradiation dose to 110 and 125 kGy is accompanied by a more noticeable change in the spectral profile, indicating the development of radiation-induced modification processes in the polymer matrix.

Thus, FTIR analysis of virgin PE-80 demonstrates that electron-beam irradiation in the range of 95–125 kGy does not destroy the core polyethylene structure but does cause its chemical modification. The preservation of the main bands of –CH_2_– groups indicates the stability of the hydrocarbon skeleton, while changes in the region of oxygen-containing groups indicate the occurrence of limited radiation-induced oxidation processes.

These changes provide a useful baseline for interpreting the more pronounced radiation-induced transformations observed in recycled polyethylene.

The spectrum of the control recycled polyethylene sample exhibits characteristic absorption bands typical of a polyethylene matrix. The most intense bands in the 2915–2920 and 2848–2850 cm^−1^ ranges correspond to asymmetric and symmetric stretching vibrations of the –CH_2_– methylene groups. The band near 1470 cm^−1^ is associated with deformation vibrations of the –CH_2_– groups, and the peaks in the 720–730 cm^−1^ range are due to the rocking vibrations of the long methylene sequences of the polyethylene chain. The presence of these bands confirms the preservation of the basic hydrocarbon structure of the polyethylene matrix in all the samples studied.

After electron-beam irradiation, no significant disappearance of the main polyethylene bands is observed, indicating the preservation of the basic structure of the polymer chain. However, changes in the shape and intensity of individual bands indicate the occurrence of radiation-induced structural transformations. Since the spectra are presented in transmittance mode (%T), a decrease in transmittance in the corresponding band region indicates an increase in absorption intensity.

For irradiated samples, changes are observed in the 1700–1740 cm^−1^ region and in the broad 3200–3500 cm^−1^ region, which are associated with oxygen-containing functional groups formed during radiation-induced oxidation processes [5,8]. The more pronounced spectral changes observed in recycled polyethylene are attributed to its initial structural heterogeneity, residual additives, products of previous thermo-oxidative degradation, and defects introduced during earlier processing.

The most pronounced changes in the spectral profile are observed for the sample irradiated at a dose of 125 kGy. In this case, changes in the region of oxygen-containing groups are enhanced, and a more noticeable change in the intensity of the main polyethylene bands is observed. This may indicate an increase in competing processes: on the one hand, electron-beam irradiation initiates the formation of macroradicals and interchain crosslinking, while on the other hand, at higher doses, the contribution of oxidative degradation and structural heterogeneity increases.

Overall, the FTIR results indicate that electron-beam irradiation preserves the fundamental polyethylene backbone while promoting chemical modification associated with the formation of oxygen-containing functional groups. The observed spectral changes indicate the simultaneous development of crosslinking and oxidation processes. The more pronounced response of recycled polyethylene is associated with its initial structural and compositional heterogeneity, which affects the balance between radiation-induced crosslinking and degradation.

### 3.2. Surface Morphology and Elemental Analysis (SEM/EDS)

**Figure 4:**
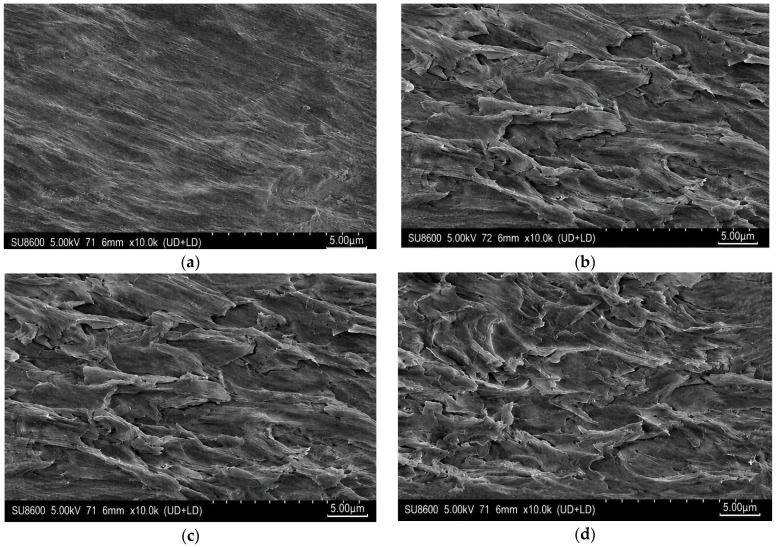
SEM micrographs of virgin PE-80 before irradiation (**a**) and after electron-beam irradiation at absorbed doses of 95 kGy (**b**), 110 kGy (**c**), and 125 kGy (**d**), recorded at ×1000 magnification.

**Figure 5:**
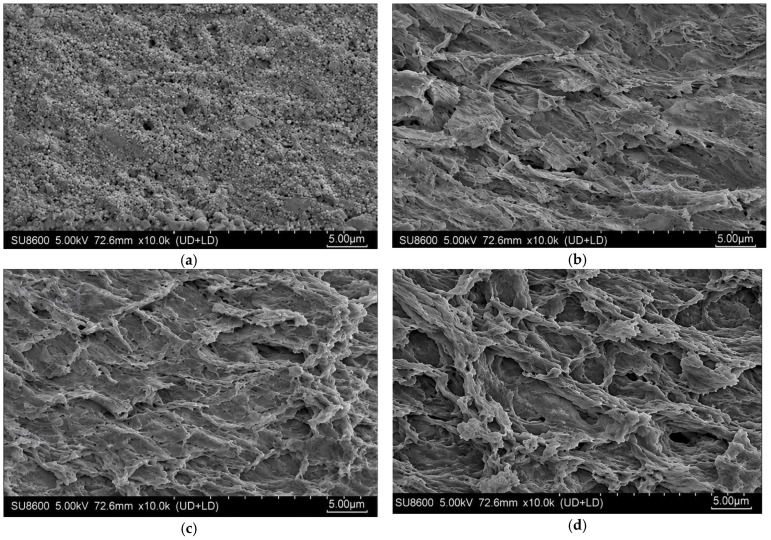
SEM micrographs of recycled polyethylene before irradiation (**a**) and after electron-beam irradiation at absorbed doses of 95 kGy (**b**), 110 kGy (**c**), and 125 kGy (**d**), recorded at ×1000 magnification.

The surface morphology and elemental composition of recycled polyethylene before and after irradiation were investigated using scanning electron microscopy (SEM) and energy-dispersive X-ray spectroscopy (EDS). SEM was used to assess irradiation-induced changes in the surface morphology of recycled polyethylene and PE-80 polyethylene. EDS analysis was employed to evaluate changes in elemental composition associated with radiation-induced oxidation and the presence of inorganic impurities. PE-80 polyethylene was analyzed under the same conditions for comparison. Figure 4 and Figure 5 present SEM micrographs of PE-80 and recycled polyethylene before and after electron-beam irradiation.

Prior to irradiation, recycled polyethylene exhibited a more heterogeneous surface morphology, including particles of different sizes and localized defective regions associated with impurities and previous processing. In contrast, virgin PE-80 showed a relatively smooth and uniform surface typical of a more regular polymer matrix. After irradiation, both materials exhibited increased surface roughness and localized microfibrillation. With increasing dose from 95 to 125 kGy, fibrillar and defective areas became more pronounced, reflecting radiation-induced structural rearrangement and crosslinking of the polymer matrix. These morphological features suggest that electron-beam irradiation modifies the failure mechanism and internal organization of the polymer network. The changes were more evident in recycled polyethylene due to its initial structural and compositional heterogeneity and are consistent with the gel fraction and mechanical testing results.

**Figure 6:**
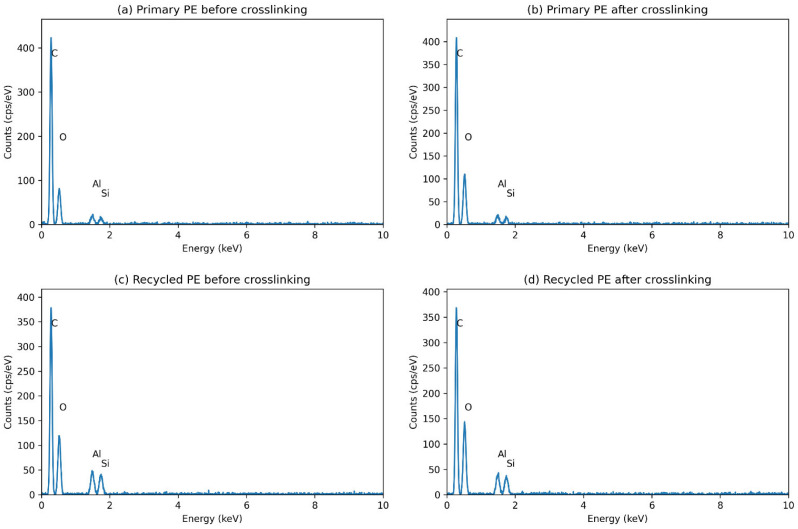
EDS spectra of virgin PE-80 and recycled polyethylene before irradiation and after electron-beam irradiation at 110 kGy: (**a**) virgin PE-80 before irradiation; (**b**) virgin PE-80 after irradiation; (**c**) recycled polyethylene before irradiation; (**d**) recycled polyethylene after irradiation.

The EDS spectra of recycled polyethylene and PE-80 polyethylene before and after irradiation at 110 kGy are shown in Figure 6. These spectra were used to evaluate changes in the elemental composition of the polymer surface layer and to identify oxygen-containing groups and inorganic impurities associated with recycled material heterogeneity.

EDS analysis was performed on samples before and after irradiation at 110 kGy, selected as a representative dose. The EDS results showed that carbon, consistent with the polyethylene matrix, was the dominant element in all samples. After irradiation, an increase in oxygen content was observed in both virgin and recycled polyethylene, indicating the occurrence of radiation-induced oxidation processes. The higher oxygen content in recycled polyethylene indicates its greater susceptibility to oxidation due to the presence of structural defects and byproducts of previous processing.

Furthermore, aluminum and silicon were detected only in the recycled polyethylene. These elements most likely originate from mineral fillers and inorganic additives commonly used in façade-fastening elements to improve stiffness, dimensional stability, and weather resistance. They may also reflect residual inorganic components remaining after previous processing and long-term service. Since the recycled polyethylene investigated in this study originated from industrial façade-fastening elements, the exact composition of the original additive package was not available. Nevertheless, the detected Al and Si are consistent with mineral fillers widely used in construction-grade polyethylene products rather than contamination introduced during sample preparation or analysis. Their presence further confirms the greater structural and compositional heterogeneity of recycled polyethylene compared with virgin PE-80.

Table 1 presents the elemental composition of the polymer samples.

Thus, the results of SEM and EDS analysis indicate that irradiation leads to both surface morphological changes and changes in the chemical composition of the polymer subsurface layer. For recycled polyethylene, these changes are more pronounced due to its initial structural and compositional heterogeneity. The obtained data are in good agreement with the results of FTIR analysis, gel fraction analysis, and mechanical testing.

### 3.3. Gel Fraction

**Figure 7:**
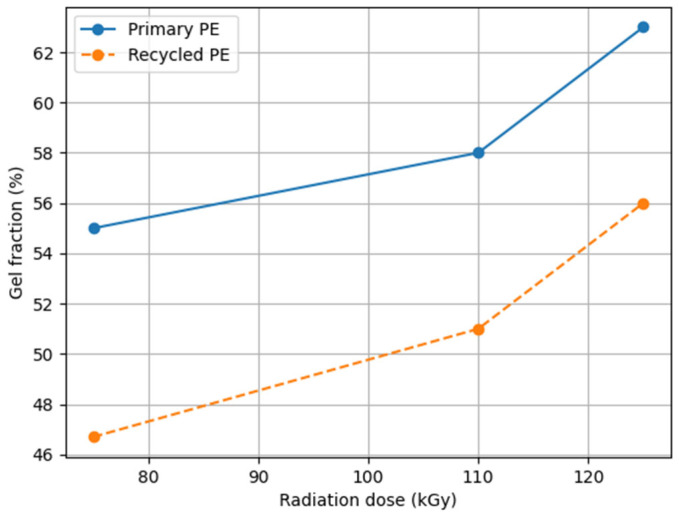
Gel fraction of primary PE-80 and recycled polyethylene as a function of irradiation dose.

The dependence of gel fraction on radiation dose for recycled polyethylene and PE-80 polyethylene is shown in Figure 7.

As shown in Figure 7, gel fraction increased monotonically with irradiation dose for both materials, confirming the progressive formation of a crosslinked network. For recycled polyethylene, gel fraction increased from 46.7 to 56.2% as the irradiation dose increased from 95 to 125 kGy, whereas virgin PE-80 exhibited higher values ranging from 55.3 to 63.4%. The higher gel fraction of virgin PE-80 reflects its more homogeneous molecular structure and lower defect content, which favor interchain crosslinking. In recycled polyethylene, residual impurities, oxidized fragments, and structural inhomogeneities increase the competition between crosslinking and chain degradation, resulting in lower gel fraction values. This behavior is consistent with previous studies reporting progressive gel fraction development in irradiated polyethylene within a similar dose range [15,42,43].

The difference between virgin and recycled polyethylene remains consistent across the entire dose range and is approximately 7–8%, confirming the lower ability to form a crosslinked structure in recycled material.

Importantly, the obtained gel fraction values are within the range reported for electron-beam-irradiated polyethylene at similar irradiation doses, where gel fraction typically reaches 50–65% in the 95–125 kGy dose range [15,42,43].

Thus, the results of the gel fraction analysis confirm that radiation exposure effectively initiates cross-linking processes in polyethylene, and the degree of cross-linking significantly depends on the initial state of the material and its structural homogeneity.

### 3.4. Thermal Properties (DSC)

**Figure 8:**
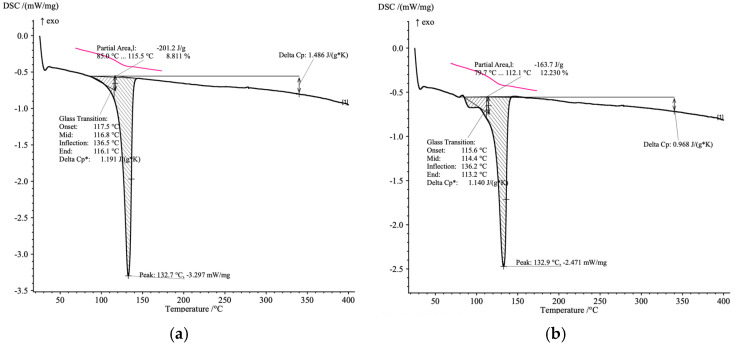
DSC heating curves of primary PE-80 (**a**) and recycled polyethylene (**b**) before irradiation. * indicates the value automatically calculated by the NETZSCH Proteus DSC software.

**Figure 9:**
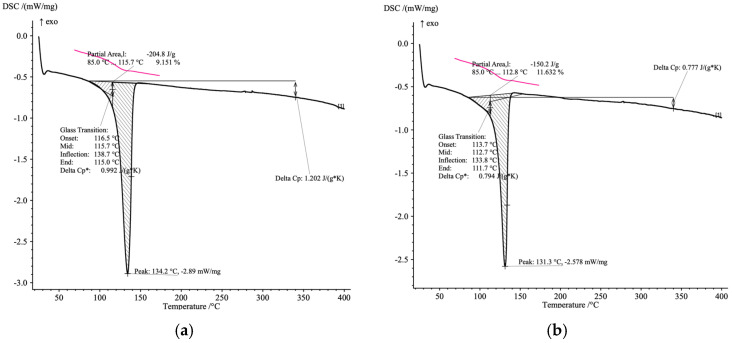
DSC heating curves of primary PE-80 (**a**) and recycled polyethylene (**b**) after electron-beam irradiation at 110 kGy. * indicates the value automatically calculated by the NETZSCH Proteus DSC software.

Differential scanning calorimetry (DSC) was used to evaluate the thermal properties of recycled polyethylene and PE-80 polyethylene before and after radiation treatment. The resulting thermograms reflect differences in the degree of structural ordering and the presence of defects in the polymer matrix. Figure 8 and Figure 9 show the DSC heating curves of PE-80 and recycled polyethylene before irradiation and after electron-beam irradiation at 110 kGy.

The main thermal parameters obtained from DSC analysis are summarized in Table 2.

As summarized in Table 2, both materials exhibited only minor changes in melting temperature after electron-beam irradiation. Virgin PE-80 showed a slight increase in melting temperature from 133.0 to 134.0 °C, whereas recycled polyethylene exhibited a slight decrease from 133.0 to 131.0 °C. More noticeable changes were observed for the melting enthalpy and degree of crystallinity. In virgin PE-80, ΔHm increased slightly from 185.5 to 189.2 J g^−1^, corresponding to an increase in crystallinity from 63.3 to 64.6%. In contrast, recycled polyethylene showed a decrease in ΔHm from 136.5 to 126.1 J g^−1^ and a reduction in crystallinity from 46.6 to 43.0%, indicating that radiation-induced crosslinking partially restricted molecular chain rearrangement and reduced the crystalline fraction. The lower crystallinity of recycled polyethylene compared with virgin PE-80 is consistent with its higher structural heterogeneity resulting from previous processing, oxidation, and the presence of residual additives.

These results agree well with the gel fraction analysis, indicating the formation of a three-dimensional crosslinked network. They are also consistent with the FTIR and SEM observations, confirming the relationship between radiation-induced structural modification and the thermal behavior of the investigated polyethylene materials.

It should be noted that DSC measurements were performed only for the initial samples and for specimens irradiated at 110 kGy. This irradiation dose was selected because it provided the most favorable balance between gel fraction development and mechanical performance and was therefore considered representative for evaluating radiation-induced changes in thermal behavior.

### 3.5. Mechanical Properties

Mechanical tests were conducted to evaluate the effect of radiation crosslinking on the strength and deformation properties of polyethylene. Analysis of the stress–strain curves revealed significant changes in material behavior after electron-beam irradiation. Tensile tests were conducted on recycled polyethylene and PE-80 polyethylene before and after irradiation at doses of 95, 110, and 125 kGy.

**Figure 10:**
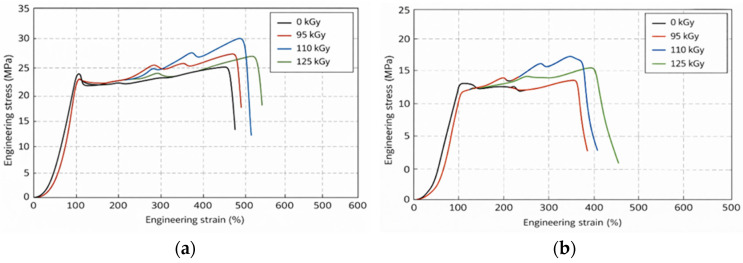
Engineering stress–strain curves of primary PE-80 (**a**) and recycled polyethylene (**b**) before and after electron-beam irradiation at doses of 95, 110, and 125 kGy.

Figure 10 shows the engineering stress–strain curves of primary PE-80 and recycled polyethylene before and after electron-beam irradiation at doses of 95, 110, and 125 kGy.

Table 3 summarizes the tensile properties of virgin PE-80 and recycled polyethylene before and after electron-beam irradiation. The quantitative results are consistent with the stress–strain curves presented in Figure 10 and demonstrate the influence of irradiation dose on the mechanical behavior of both materials.

For virgin PE-80, electron-beam irradiation increased both tensile strength and elastic modulus, with the highest values obtained at 110 kGy. Although elongation at break remained relatively high over the investigated dose range, further irradiation to 125 kGy resulted in a decrease in tensile strength, indicating the onset of competing degradation processes.

Recycled polyethylene exhibited lower tensile strength than virgin PE-80 because of its greater structural and compositional heterogeneity. Nevertheless, electron-beam irradiation noticeably improved its mechanical performance. The tensile strength increased from 12.04 MPa for the non-irradiated material to 17.56 MPa at 110 kGy, while the elastic modulus also reached its maximum value (453 MPa). At 125 kGy, both tensile strength and elastic modulus decreased despite a slight increase in elongation at break, suggesting that radiation-induced chain scission increasingly competes with crosslinking at higher irradiation doses.

Considering the mechanical results together with the gel fraction, FTIR, SEM, and DSC analyses, the absorbed dose of 110 kGy provides the most favorable balance between radiation-induced crosslinking and preservation of the mechanical performance of recycled polyethylene.

Analysis of the stress–strain curves indicates that electron-beam irradiation significantly modifies the tensile deformation behavior of polyethylene, particularly during the post-yield deformation stage. While the yield stress changes only slightly, irradiation results in reduced elongation at break and suppression of the cold-drawing stage, accompanied by more pronounced strain hardening. These changes indicate the formation of a three-dimensional cross-linked network that restricts molecular chain mobility and reduces the ductility of the material. Similar changes in the mechanical behavior of electron-beam-irradiated polyethylene have been reported in previous studies, where radiation-induced crosslinking increased stiffness and strength while progressively limiting plastic deformation and molecular chain orientation during tensile loading [15,42].

The obtained results are also consistent with the SEM observations revealing morphological changes after irradiation and with the DSC analysis showing modifications in crystallinity. Previous studies have likewise demonstrated that the mechanical performance of electron-beam-irradiated polyethylene is closely related to changes in crosslink density and crystalline structure, confirming that the final properties are governed by the balance between radiation-induced crosslinking and chain degradation processes [42].

Overall, the mechanical results confirm that electron-beam irradiation improves the strength and stiffness of both virgin and recycled polyethylene through radiation-induced crosslinking. Similar increases in stiffness accompanied by reduced ductility have been reported for electron-beam-irradiated polyethylene and are commonly attributed to the formation of a crosslinked network that restricts molecular chain mobility [15,42]. However, excessive irradiation promotes competing degradation processes, as indicated by the decrease in tensile strength and elastic modulus at 125 kGy. Among the investigated doses, 110 kGy provided the most favorable balance between crosslinking-induced strengthening and preservation of deformation capacity, particularly for recycled polyethylene. Therefore, the obtained results are consistent with the behavior of electron-beam-irradiated polyethylene reported in the literature and confirm the applicability of radiation crosslinking for improving the performance of recycled polyethylene.

## 4. Discussion

The obtained results demonstrate that electron-beam irradiation initiates significant structural transformations in recycled polyethylene, affecting its chemical structure, morphology, thermal behavior, and mechanical properties. The observed changes are governed by the competition between radiation-induced crosslinking and degradation processes, which is widely recognized as a key mechanism controlling the response of polyethylene to ionizing radiation [11,15].

FTIR analysis results confirm that after irradiation, the main polyethylene bands associated with vibrations of the –CH_2_– methylene groups are retained in both virgin PE-80 and recycled polyethylene. This indicates the preservation of the basic hydrocarbon structure of the polyethylene matrix after electron beam treatment. At the same time, changes in the spectral regions of 1700–1740 cm^−1^ and 3200–3500 cm^−1^ indicate the formation of oxygen-containing functional groups associated with radiation-induced oxidation processes [5,8,38].

Comparison of the FTIR spectra of PE-80 and recycled polyethylene shows that the spectral changes associated with oxygen-containing functional groups are more pronounced in the recycled material. This is due to the initial structural and compositional heterogeneity of recycled polyethylene, the presence of products of previous thermal-oxidative degradation, residual additives, impurities, and structural defects. These features contribute to more active oxidative and destructive processes during irradiation. In contrast, PE-80 exhibits less pronounced spectral changes, which reflects its higher structural homogeneity and lower susceptibility to radiation-induced oxidation.

An increase in gel fraction with increasing irradiation dose confirms the formation of a spatially cross-linked network structure [44]. Higher gel fraction values for virgin PE-80 indicate greater efficiency of interchain cross-linking in a more homogeneous polyethylene matrix. For recycled polyethylene, the increase in gel fraction from 46.7 to 56.2% confirms effective formation of a crosslinked network despite the structural heterogeneity of the material. The lower gel fraction values compared with PE-80 indicate that part of the generated macroradicals participates in oxidation and degradation reactions in addition to crosslinking processes. This interpretation is consistent with the FTIR results showing more pronounced formation of oxygen-containing groups in the recycled material.

These observations are in good agreement with previous studies reporting that electron-beam irradiation of polyethylene produces a progressive increase in gel fraction due to the formation of an insoluble three-dimensional network. Similar dose-dependent behavior has been described for irradiated polyethylene in the dose range investigated in the present work [15,42].

The EDS analysis results further confirm the FTIR spectroscopy data. After irradiation, an increase in oxygen content is observed in both virgin and recycled polyethylene, but this effect is more pronounced in the recycled material. Furthermore, the presence of aluminum and silicon in the recycled polyethylene indicates the presence of mineral fillers or process impurities, confirming its higher compositional heterogeneity. Such heterogeneity may facilitate the localization of radiation-induced processes and exacerbate the differences in the behavior of virgin and recycled materials.

The morphological changes revealed by SEM are also consistent with the results of FTIR, EDS, and gel fractionation analysis. After irradiation, both materials exhibit an increase in surface roughness and the formation of fibrillar and defective areas. These changes reflect the reorganization of the polymer matrix as a result of the formation of a spatially cross-linked structure and a change in the nature of material failure [45]. For recycled polyethylene, morphological changes are more pronounced, which is associated with its initial structural heterogeneity and more active competing processes of crosslinking, oxidation, and degradation. Similar irradiation-induced morphological changes, including increased surface roughness and defect formation, have also been reported for electron-beam-modified polyethylene and are commonly associated with simultaneous crosslinking and localized degradation processes [13,42].

DSC data confirm a change in the ratio of crystalline to amorphous phases after irradiation. The decrease in crystallinity, particularly in recycled polyethylene, may be due to disruption of the regular packing of macromolecular chains due to crosslinking, defect accumulation, and destructive processes [41]. In virgin PE-80, thermal changes are less pronounced, consistent with its higher structural homogeneity and more efficient formation of a crosslinked network. Similar reductions in crystallinity after electron-beam irradiation have also been reported previously and are generally attributed to restricted chain rearrangement caused by network formation [15,18].

Mechanical properties reflect the combined effect of the identified structural changes. The increase in strength and elastic modulus after irradiation is associated with the formation of a spatially crosslinked structure, which limits the mobility of macromolecular chains. At the same time, a decrease in relative elongation at break indicates a decrease in ductility and a transition to a more rigid deformation behavior of the material. For recycled polyethylene, the decrease in ductility is more pronounced, due to a combination of crosslinking, structural heterogeneity, and radiation-induced degradation.

The combined characterization results establish a clear structure–property relationship for both virgin and recycled polyethylene. The observed changes in chemical structure, gel fraction, morphology, and crystallinity are directly reflected in the mechanical performance of the irradiated materials. This integrated structure–property relationship represents the principal scientific contribution of the present study and demonstrates that controlled electron-beam irradiation can effectively upgrade structurally heterogeneous recycled polyethylene for higher-value applications.

Overall, the obtained results indicate that an absorbed dose of 110 kGy provides the most favorable balance between crosslink formation and preservation of mechanical performance. Similar optimum irradiation ranges have been reported previously for polyethylene, where moderate radiation doses maximize the beneficial effects of crosslinking, whereas further dose increases enhance oxidative degradation and chain scission [13,15,42].

This conclusion is supported by the combined evaluation of all characterization methods employed in this study. At an absorbed dose of 110 kGy, recycled polyethylene exhibited a high gel fraction, only moderate changes in crystallinity, limited formation of oxygen-containing functional groups, and the highest tensile strength and elastic modulus without excessive degradation. Although increasing the irradiation dose to 125 kGy resulted in a further increase in crosslink density, it also intensified oxidation and chain degradation, leading to reduced ductility and limiting further improvement of the overall mechanical performance. These results demonstrate that, beyond the optimum irradiation dose, degradation processes progressively compete with radiation-induced crosslinking and become the dominant factor controlling the final properties of recycled polyethylene [5,38].

Thus, electron-beam irradiation can be considered an effective approach for upgrading recycled polyethylene. Despite its greater structural and compositional heterogeneity, recycled polyethylene forms a crosslinked network structure and demonstrates improved strength and stiffness after irradiation. The results indicate that an irradiation dose of 110 kGy provides a favorable balance between crosslinking efficiency and preservation of ductility, making electron-beam irradiation a promising approach for enhancing the performance and expanding the application potential of recycled polyethylene products.

From a practical perspective, electron-beam irradiation represents a promising technology for upgrading recycled polyethylene, particularly for applications requiring improved mechanical performance without the use of chemical crosslinking agents. Although electron accelerators require considerable capital investment, the operating cost per processed product is relatively low because irradiation is completed within seconds, does not require chemical initiators or post-curing, and can be readily integrated into continuous industrial production lines [11,15,23]. Compared with conventional peroxide crosslinking, electron-beam processing provides precise control of the absorbed dose, eliminates residual crosslinking by-products, and offers high process reproducibility, making it attractive for manufacturing high-value polymer products [3,11,15].

The industrial applicability of this technology depends on product geometry and wall thickness. The specimens investigated in the present study (approximately 2 mm thick) were well within the effective penetration capability of the 5 MeV electron beam, ensuring uniform irradiation throughout the entire specimen thickness. For thicker polyethylene products, uniform crosslinking may require double-sided irradiation or the use of higher-energy electron accelerators to achieve a more homogeneous absorbed dose distribution throughout the material [11,23]. Therefore, electron-beam crosslinking can be considered a practical industrial technology for continuous processing of recycled polyethylene products with controlled dimensions and high production throughput, making it a promising approach for enhancing the performance and expanding the application potential of recycled polyethylene.

## 5. Conclusions

In the present study, the effect of electron-beam irradiation on the structure and properties of recycled polyethylene obtained from façade-fastening elements was systematically investigated using virgin PE-80 polyethylene as the reference material. The results demonstrated that electron-beam irradiation effectively modifies the recycled polymer through the simultaneous development of crosslinking, oxidation, and structural rearrangement processes.

The combined results of FTIR, SEM/EDS, DSC, gel fraction analysis, and mechanical testing established a clear structure–property relationship linking radiation-induced structural evolution with the resulting thermal and mechanical behavior of the investigated materials. FTIR and EDS analyses indicated radiation-induced oxidation accompanied by an increase in oxygen content, while SEM observations revealed the development of a more heterogeneous and fibrillar surface morphology. DSC analysis confirmed a decrease in crystallinity of recycled polyethylene associated with network formation during irradiation.

Despite its greater structural and compositional heterogeneity, recycled polyethylene exhibited effective radiation-induced crosslinking. The gel fraction increased from 46.7 to 56.2%, while the tensile strength increased from 12.04 to 17.56 MPa and the elastic modulus from 268 to 453 MPa. These results demonstrate that electron-beam irradiation significantly improves the mechanical performance of recycled polyethylene despite its initially heterogeneous structure.

Among the investigated irradiation doses, 110 kGy provided the most favorable balance between crosslinking efficiency and preservation of mechanical performance. Overall, the obtained results demonstrate that controlled electron-beam irradiation is an effective approach for upgrading recycled polyethylene and expanding its potential for reuse in higher-performance applications within the framework of a circular economy.

## Figures and Tables

**Table 1:** Elemental composition of polymer samples.

Element	Atomic Number	Primary PE Before Irradiation, at.%	Primary PE After Irradiation at 110 kGy, at.%	Recycled PE Before Irradiation, at.%	Recycled PE After Irradiation at 110 kGy, at.%
C	6	97.85	96.94	94.32	93.48
O	8	1.62	2.54	3.12	4.02
Al	13	0.33	0.31	1.34	1.30
Si	14	0.20	0.21	1.22	1.20
Total		100.00	100.00	100.00	100.00

**Table 2:** Thermal parameters obtained from DSC analysis of virgin PE-80 and recycled polyethylene before and after electron-beam irradiation.

Sample	Irradiation Dose (kGy)	Tm (°C)	ΔHm (J g^−1^)	Xc (%)
PE-80	0	133.0	185.5	63.3
PE-80	110	134.0	189.2	64.6
Recycled polyethylene	0	133.0	136.5	46.6
Recycled polyethylene	110	131.0	126.1	43.0

**Table 3:** Tensile properties of virgin PE-80 and recycled polyethylene before and after electron-beam irradiation.

Material	Dose (kGy)	Tensile Strength, MPa	Elongation at Break, %	Elastic Modulus, MPa
Virgin PE-80	0	25.0	480	405
Virgin PE-80	95	28.2	490	426
Virgin PE-80	110	30.0	510	452
Virgin PE-80	125	26.4	530	435
Recycled polyethylene	0	12.04	360	268
Recycled polyethylene	95	13.78	390	435
Recycled polyethylene	110	17.56	350	453
Recycled polyethylene	125	15.09	440	408

## Data Availability

The original contributions presented in this study are included in the article. Further inquiries can be directed to the corresponding authors.
